# Findings from the initial Stepwise Approach to Rabies Elimination (SARE) Assessment in China, 2019

**DOI:** 10.1371/journal.pntd.0009274

**Published:** 2021-03-29

**Authors:** Qiulan Chen, Xiaoyue Ma, Jeanette J. Rainey, Yu Li, Di Mu, Xiaoyan Tao, Ye Feng, Wenwu Yin, Zhongjie Li, Shichun Ma, Brett Petersen

**Affiliations:** 1 Division of Communicable Diseases, Chinese Center for Disease Control and Prevention, Beijing, China; 2 Division of High-Consequence Pathogens and Pathology, United States Centers for Disease Control and Prevention, Atlanta, United States of America; 3 Division of Global Health Protection, United States Centers for Disease Control and Prevention, Beijing, China; 4 National Institute for Viral Disease Control and Prevention, Chinese Center for Disease Control and Prevention, Beijing, China; 5 Institute of Military Veterinary Medicine, Academy of Military Medical Sciences, National Reference Laboratory for Animal Rabies, Changchun, China; 6 Division of Public Health, China Animal Disease Control Center, Beijing, China; University of Surrey, UNITED KINGDOM

## Abstract

In 2015, China and other member states of the United Nations adopted the goal of eliminating dog-mediated rabies by 2030. China has made substantial progress in reducing dog-mediated human rabies since peaking with more than 3,300 reported cases in 2007. To further improve coordination and planning, the Chinese Center for Disease Control and Prevention, in collaboration with the United States Centers for Disease Control and Prevention, conducted a Stepwise Approach towards Rabies Elimination (SARE) assessment in March 2019. Assessment goals included outlining progress and identifying activities critical for eliminating dog-mediated rabies. Participants representing national, provincial and local human and animal health sectors in China used the SARE assessment tool to answer 115 questions about the current dog-mediated rabies control and prevention programs in China. The established surveillance system for human rabies cases and availability of post-exposure prophylaxis were identified as strengths. Low dog vaccination coverage and limited laboratory confirmation of rabid dogs were identified gaps, resulting in an overall score of 1.5 on a scale of 0 to 5. Participants outlined steps to increase cross-sectoral information sharing, improve surveillance for dog rabies, increase dog vaccination coverage, and increase laboratory capacity to diagnose rabies at the provincial level. All assessment participants committed to strengthening cross-sector collaboration using a One Health approach to achieve dog-mediated human rabies elimination by 2030.

## Author summary

Rabies is an acute zoonosis with a mortality rate of almost 100% following the onset of symptoms. In China, the increased access to post-exposure prophylaxis has substantially reduced the number of human rabies deaths. However, rabies continues to circulate in many rural parts of the country, resulting in hundreds of rabid dog-related exposures and deaths each year. In 2015, member states of the United Nations adopted the goal of eliminating dog-mediated rabies by 2030. We conducted a Stepwise Approach towards Rabies Elimination (SARE) assessment to outline progress and identify steps needed to achieve the elimination goal in China. The assessment involved representatives from the national and 12 selected provincial-level human and animal health sectors. The 12 provinces were purposefully selected based on the current dog-mediated rabies burden, an interest in participating, and the ability to complete the SARE assessment tool. This cross-sector–or One Health—approach was useful for improving coordination and planning. In particular, the assessment identified opportunities to improve collaboration between sectors on laboratory diagnostic capacity, implementing mass dog vaccination campaigns and conducting epidemiologic investigations on dog-mediated rabies exposures. An integrated One Health strategy should be strengthened in China as well as in other countries aiming to eliminate dog-mediated rabies.

## Introduction

Rabies is a zoonotic disease that causes approximately 59,000 human deaths globally each year [1,2]. Of these deaths, 99% result from exposures to rabid dogs [1,3]. Primary interventions focus on increasing rabies vaccination coverage through routine veterinarian services and mass dog vaccination campaigns. Eliminating the rabies virus among the dog population typically requires achieving and maintaining coverage of 70% for at least 3–7 years [4–6]. Strengthening management of the dog population and investigations of suspect rabies animals and possible rabies exposures are also essential components of a rabies control and prevention program. Although almost always fatal following the onset of symptoms, human deaths can be prevented by secondary interventions such as the appropriate and timely use of post exposure prophylaxis (PEP).

The World Health Organization (WHO), World Organisation for Animal Health (OIE), the Food and Agriculture Organisation (FAO) and the Global Alliance for Rabies Control (GARC) proposed a goal of eliminating dog-mediated rabies by 2030. China and other member states of the United Nations agreed to adopt this goal in 2015 [7]. China, along with Japan and South Korea, and the Association of South East Asian Nations (ASEAN) also developed a regional strategic plan for dog-mediated rabies control in 2016. The regional plan has focused on establishing coordination mechanisms between countries and stakeholders, strengthening capacities of the Veterinary Services and Human Health Services, and encouraging high-level governmental engagement [8]. At the national level, the Chinese government has prioritized rabies vaccines for dogs, supported investigation and quarantine of biting dogs, halted the production and use of live rabies vaccine for animals [9–11], and promoted rabies prevention education and awareness programs, particularly in high-risk provinces. The China national reference laboratory for animal rabies has provided training to more than 500 laboratory staff from provincial and municipal animal disease control centers (ACDC), and the OIE reference laboratory in China has recently provided training to more than 60 laboratory staff from other ASEAN countries.

To further improve coordination and planning needed to advance the ASEAN strategic plan and achieve the 2030 elimination goal, the Chinese Center for Disease Control and Prevention (China CDC) collaborated with the United States Centers for Disease Control and Prevention (US CDC) to conduct a Stepwise Approach towards Rabies Elimination (SARE) assessment in March 2019. Anticipated outcomes included initial steps for developing a national strategic plan and identifying mechanisms to support and monitor progress in terms of rabies control and prevention activities using a cross-sector One Health approach. Here, we describe the current dog-mediated rabies control activities in China, the SARE assessment methodology, the outcome of the SARE assessment, and the cross-sector plans and activities to achieve dog-mediated rabies elimination.

## Methods

### Ethics statement

China CDC approved the SARE assessment as a program evaluation activity that relied on routine public health and animal health surveillance data as well as publicly available data, and therefore was exempt from institutional review board assessment.

### SARE tool

The Stepwise Approach towards Rabies Elimination (SARE) assessment tool was jointly developed by FAO, GARC and WHO to support rabies-endemic countries in the development and implementation of their own sustainable rabies elimination strategy. The tool provides an expert-reviewed and non-prescriptive six-stage roadmap, ranging from ‘Stage 0’–no information on rabies, to ‘Stage 5’–valid and timely data confirming rabies elimination [12]. The tool is divided into seven categories which align with the Global Framework for the Elimination of Dog-Mediated Human Rabies (STOP-R framework) [13] and the ASEAN Strategic Plan. The categories include the following.

1. Rabies Information, Education, and Communication–advocacy initiatives implemented in country or province;
2. Dog population related issues–dog population size, turnover, and management practices;
3. Prevention and control–existing rabies intervention approaches in place (e.g., availability and use of dog vaccine, biologics and human PEP);
4. Data collection and analysis–existing surveillance network and epidemiologic analysis of rabies data;
5. Laboratory diagnosis–diagnostic capacity for human and animal rabies at national and provincial level;
6. Cross-cutting issues–collaboration across human and animal health sectors and other stakeholders;
7. Legislation–existing legislation on rabies control and elimination in country or province.

Assessment participants from human and animal sectors answer either “yes” or “no” to a set of 115 general and technical questions across the seven categories listed above. These answers are represented as 1 or 0 and totaled across the seven categories. The SARE score calculated from this total indicates the stage of progress towards rabies elimination.

Stage 0: No information on rabies is available, but rabies is suspected to be present
Stage 1: Assessment of local rabies epidemiology, elaboration of a short-term rabies action plan
Stage 2: Development of a national rabies prevention and control strategy
Stage 3: Full-scale implementation of the national rabies control strategy
Stage 4: Maintenance of human rabies freedom, elimination of dog rabies
Stage 5: Freedom from human and dog rabies, ongoing monitoring and evaluation

Each stage includes multiple essential and non-essential activities. Although not all the activities are mandatory, the essential activities determine whether a country can advance to the next stage. The non-essential activities provide additional guidance for evaluating progress within a specific stage towards the goal of eliminating dog-mediated rabies.

### SARE assessment activities and next step planning

The SARE assessment tool and related materials were translated into Chinese for the assessment. Assessment participants included 33 representatives from national- and 12 provincial-level human and animal health sectors (**Table 1**). The 12 provincial-level teams were selected according to the current human rabies case burden (high, low and moderate), interest in participating in the assessment, and ability to aggregate the data needed to complete the assessment tool. The 33 representatives were divided into 13 teams–one for the national rabies programs and 12 individual teams for each province participating in the assessment.

**Table 1:** Agencies* participating in the Stepwise Approach towards Rabies Elimination (SARE) assessment, Beijing, China, March 2019.

National agencies Chinese Center for Disease Control and Prevention (China CDC) China Animal Disease Control Center (China Animal CDC) Institute of Military Veterinary Medicine, Academy of Military Medical Sciences (IMVM) Changchun Veterinary Research Institute, Chinese Academy of Agriculture Sciences (CVRI) Chinese Center for Health Education. High-burden provinces in China Hunan Provincial CDC Hunan Provincial Animal CDC Guangxi Autonomous Regional CDC Guangxi Autonomous Regional Animal CDC Guizhou Provincial CDC Guizhou Provincial Animal CDC Guangdong Provincial CDC Guangdong Provincial Animal CDC Yunnan Provincial CDC Yunnan Provincial Animal CDC Moderate-burden provinces in China Anhui Provincial CDC Anhui Provincial Animal CDC Chongqing Municipal CDC Chongqing Municipal Animal CDC Hebei Provincial CDC Hebei Provincial Animal CDC Hubei Provincial CDC Hubei Provincial Animal CDC Henan Provincial CDC Henan Provincial Animal CDC Shandong Provincial CDC Shandong Provincial Animal CDC Low-burden provinces in China Beijing Municipality CDC Beijing Municipality Animal CDC
International Organizations United States Centers for Disease Control and Prevention (US CDC) United States Department of Agriculture (USDA) World Health Organization (WHO) Food and Agriculture Organization of the United Nations (FAO) World Organisation for Animal Health (OIE) World Animal Protection

*A total of 33 representatives from national and provincial level agencies completed the SARE Tool. Burden is categorized by number of human rabies cases reported in 2018. Chongqing and Beijing are Municipalities directly under the Central Government and administratively report directly to the Central Government. CVRI serves as the national reference laboratory for animal rabies and was certificated by OIE in 2012.

Before starting the SARE assessment, representatives from each of the 13 teams introduced the status of human and animal rabies and current prevention and control activities for their respective national or provincial level program. Input for several provincial level scoring criteria relied on national level information, particularly for legislation and vaccine availability. The 13 teams completed the tool collaboratively with guidance from US CDC staff. This process generated national and individual provincial level scores. Due to similar findings across the provincial-level rabies programs, assessment participants collaborated to outline goals and priority activities for each of the seven SARE categories. National and provincial-level representatives were divided into seven three- to five-member groups to outline the next steps for addressing the identified gaps for each category. An international rabies consultant was assigned to each team to help guide discussions and provide examples from other countries.

## Results and discussion

### Current rabies control activities in China

**Fig 1:**
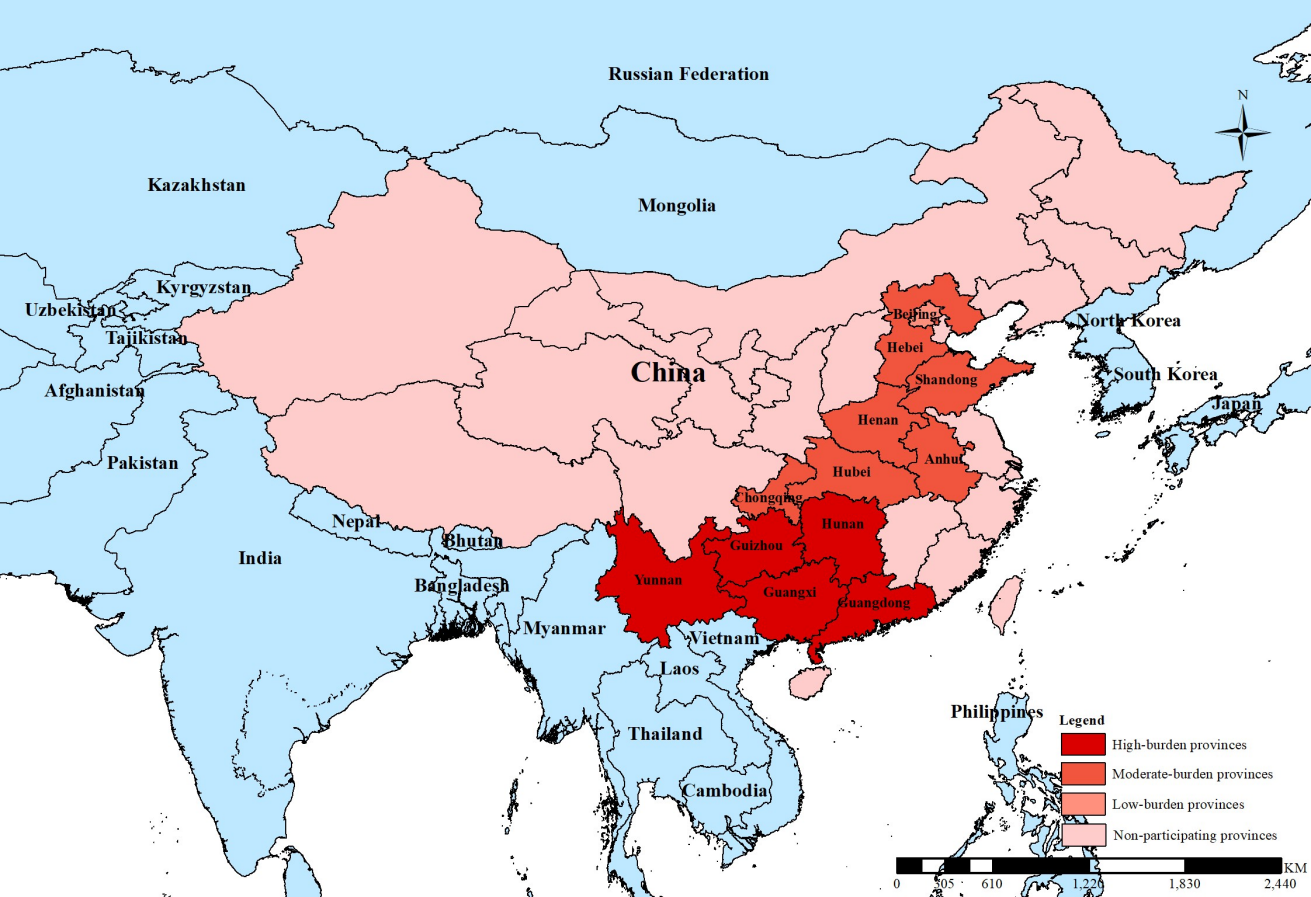
Location of the 12 provinces participating in the SARE Workshop by dog-mediated rabies burden, March 2019, China. (Note: China CDC and China Animal CDC offices are located in Beijing. SARE referred to Stepwise Approach towards Rabies Elimination).

Human rabies has been a notifiable disease in China since the early 1950s. Since 2003, all human cases have been reported to the national notifiable disease surveillance reporting system (NNDRS). Each suspected human rabies case is investigated by county-level Center for Disease Control (CDCs) and reported directly to NNDRS which is maintained by China CDC in Beijing. China has made substantial progress in reducing human rabies since peaking with more than 3,000 reported cases in 2007. The mean number of annually reported human rabies cases declined to 661 between 2014 and 2018. This is significantly lower than global burden estimates from 2015, suggesting that 6,002 (95% CI: 1,000–11,000) human rabies cases may occur in China each year [1]. The majority of dog-mediated rabies cases occur in the rural areas of five high-risk provinces in Southern China–Hunan, Guangdong, Guangxi, Guizhou, and Yunnan [14,15] (**Fig 1**), and less than 2% of human cases are laboratory confirmed. The difference between reported cases and disease burden estimates, therefore, may reflect methodologic considerations (eg. age of some of the data sources used to generate these estimates from ~2010) and actual under-reporting of rabies in certain areas of the country.

Health care for potential rabies exposures, including PEP, is available at county-level CDCs and township-based rabies clinics, and is administered according to recommendations and wound categories defined by WHO [16]. PEP vaccination series can be administered using either the Zagreb 2-1-1 or the five-dose Essen regimens. Rabies immune globulin is provided for WHO category III exposures (i.e., single or multiple transdermal bites or scratches, contamination of mucous membrane or broken skin with saliva from animal licks, exposures due to direct contact with bats). Both human rabies immune globulin (HRIG) and equine rabies antiserum (ERA) are approved in China. The Chinese National Medical Products Administration review and approve the use of human rabies vaccines and rabies immune globulin. The full PEP series is currently paid by the patient or patient’s family as an “out of pocket” cost of approximately $US48.

The Department of Public Security (DPS) is responsible for management of the dog population in urban areas. Eight provinces and more than 70 cities have issued additional regulations on dog management. Beijing Municipality, for example, has issued a regulation limiting each household to only one dog [17]. DPS also captures or collects unlicensed, biting, wild or dead dogs identified in urban areas. Dog rabies has been a nationally notifiable animal disease since the early 1950’s. According to National Animal Rabies Prevention and Control Plan, biting dogs showing rabies-like clinical signs should be evaluated by the local ACDC as suspected rabid dogs [10]. These dogs should be tested and confirmed for rabies by the national reference laboratory for animal rabies. Provincial ACDCs submit monthly reports on suspected and confirmed dog rabies to the Animal Disease Surveillance System (ADSS), which is maintained by the China Animal CDC in Beijing. In 2017, 65 rabid dogs were reported to ADSS [18]. The majority of these dogs were reported as suspected rather than laboratory confirmed rabies. WHO estimates that China is home to approximately 28 million domestic, stray and free-roaming dogs [3].

The Department of Animal Husbandry and Veterinary Medicine in the Ministry of Agriculture and Rural Affairs are responsible for the provision of dog rabies vaccinations. These responsibilities include supplying rabies vaccine for veterinary use, administering dog rabies vaccine, recording immunization history and issuing of "domestic dog rabies immunization certificate", and monitoring rabies outbreaks among dogs. Dog owners are required by law to register pets at the local Department of Public Security annually, after which dogs are vaccinated against rabies [10,19]. The cost of annual dog registration (which includes the rabies vaccination) varies by province and can range from $28 to $140. Five international and ten domestically manufactured animal rabies vaccines are available for use in China. The China Institute of Veterinary Drugs Control (IVDC) is responsible for reviewing and approving the use of animal vaccines. According to records maintained by the Department of Public Security, approximately 40% of domestic (owned) dogs are vaccinated against rabies.

### SARE assessment outcomes

**Fig 2:**
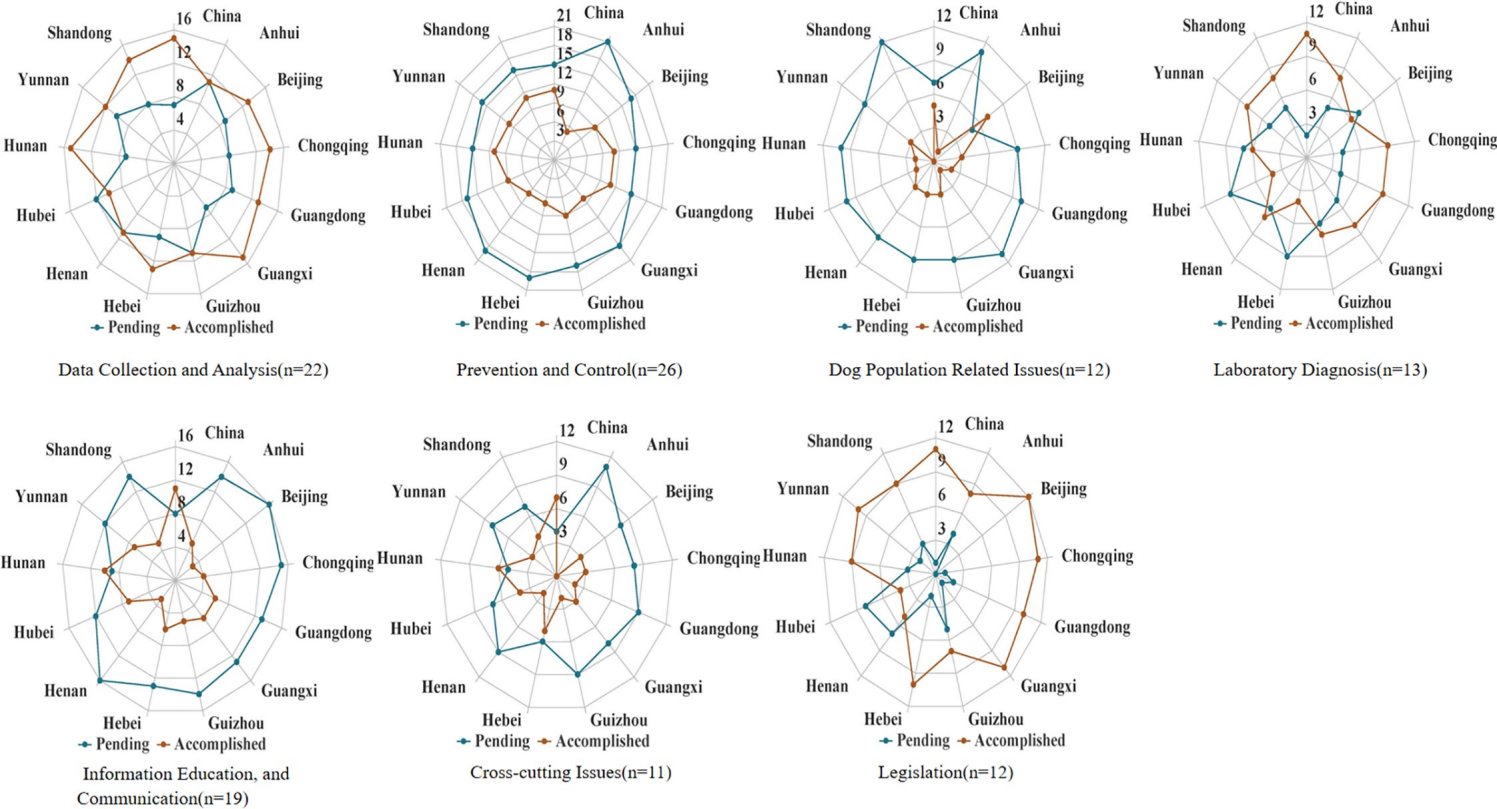
Summary of results for the National Rabies Program and for 12 Provincial level Programs, by the seven categories outlined in the SARE assessment tool, China, March 2019. (Note: For each category, the number of pending and accomplished activities are presented. SARE referred to Stepwise Approach towards Rabies Elimination).

Results for each of the seven assessment categories are presented in **Fig 2** and **S1 Table**. National and provincial level programs have accomplished the greatest progress in data collection and analysis, legislation and capacity to perform laboratory diagnosis for rabies. At the same time, the prevention and control and dog population management components had the largest number of pending activities and require the most attention. Based on these accomplishments and pending activities, China received a SARE score of 1.5 out of 5. Eleven of the 12 provinces also received a score of 1.5. The exception was for Hubei Province, with a score of 0.5. This lower score was primarily due to low laboratory capacity. Low dog vaccination coverage, lack of Integrated Bite Case Management (IBCM) at local level, lack of national strategy for dog population management (DPM), no baseline number of dogs, lack of effective inter-sectoral rabies task force at any level contributed to the low SARE scores at both the national and provincial level (**Table 2**). A qualitative description of the SARE results is included below.

1. Collection and analysis of data:
  National surveillance systems for human and animal rabies have been established in China. Although these systems operate separately, data are analyzed on a regular basis. Feedback is provided to provincial and local level rabies programs through an annual rabies report. National support is provided to local and provincial CDCs to conduct epidemiologic investigations of human cases and to monitor use of PEP in high-risk regions. Gaps were identified in animal rabies case reporting (the number of dog rabies was far below the number of human cases). Additionally, information sharing between the human and animal health sectors was infrequent.
2. Prevention and control:
  Human rabies prevention has focused primarily on increasing availability and access to PEP following potential rabies exposures. Chinese manufacturers provide rabies vaccine for domestic use. Because the vaccine meets international standards, the manufacturers have applied for vaccine pre-certification from the WHO. China recently issued the Rabies Post Exposure Prophylaxis Guidelines (version 2009) [20] and the Rabies Prevention and Control Technical Guidelines (version 2016) [21] to standardize rabies PEP practices. These documents incorporate information included position papers and technical reports published by WHO [14,22]. The department of health has modified the medical insurance system, including implementation of the New Rural Cooperative Medical System, to allow for greater reimbursement for PEP related health care expenses in some provinces such as Guizhou, Hunan and Guangxi [23]. This health system’s approach has increased the quality and affordability of the PEP, especially in the rural areas of these high-risk provinces, and has contributed to the observed decline of human rabies cases.
  Controlling and preventing rabies in the dog population remains limited in China, partially due to the lack of uniform policies and guidelines as well as facilities for dog observation (following a bite event). Dog rabies vaccination is provided with pet registration and coverage is generally high in urban settings (e.g., above 70% in Shanghai and in the Pearl River Delta region in Guangdong Province). However, dog registration and vaccination in rural areas are weakly managed and vaccination coverage remains low. The Red-Collar Program, a pilot dog vaccination campaign project, was implemented in three counties in Guizhou, Anhui, and Shanxi Provinces [24]. The campaign achieved 92% vaccination coverage among domestic (owned) dogs. China has not yet implemented a large-scale mass dog vaccination campaign targeting stray and free-roaming dogs.
3. Controlling the number of dogs:
  China’s public security sector is responsible for the registration of dogs. Dog registration and management has greatly improved in urban settings [25,26]. Several non-governmental organizations have been established to collaborate with government agencies in providing shelter and management of stray dogs [27,28]. The lack of national and provincial level strategies and plans for dog population control was a major gap identified in the SARE assessment. During previous public emergencies (clusters of dog bites), dog population was reduced by euthanizing identified stray and free-roaming dogs. China has not yet implemented a dog census, and information on the number of both domestic and free-roaming dogs is currently based on WHO generated estimates [3].
4. Laboratory diagnosis:
  The national laboratory at China CDC, Beijing is responsible for diagnosis of human rabies using tests recommended by WHO, including the Direct Fluorescent Antibody (DFA) Test, Immunohistochemistry (IHC) Test, Direct Rapid Immunohistochemistry Test (DRIT), Real Time RT-PCR, virus isolation and rabies virus variant typing. This capacity also exists at the provincial level and in certain prefecture-level CDC laboratories. Because of challenges in obtaining specimens from human cases, less than 2% of all reported human rabies cases are laboratory confirmed. On animal side, the national and OIE reference laboratory for animal rabies, designated by Ministry of Agriculture and Rural Affairs of China and OIE respectively, is responsible for diagnosis of animal rabies at national level, but the laboratory capacity at the local levels remains weak.
5. Health promotion and rabies prevention education:
  Health promotion and rabies prevention education activities are conducted annually at the national and provincial level on World Rabies Day [23]. The National 12320 Health Hotline Management Office and the China Center for Health Education have developed and distributed rabies education material through social media channels (Weibo and WeChat). Other rabies prevention and control promotion material has been shared through national and local newspaper outlets. However, none of the provincial level representatives had developed strategies or plans for the systematic or continuous implementation of rabies prevention and education activities. Most current rabies education materials have not yet been adapted to target different socio-economic, age, and ethnic at-risk population in China.
6. Cross-department cooperation:
  During public emergencies, such as numerous bites or wounds caused by single or multiple dogs in a specific community, human and animal staff will meet on an ‘ad-hoc’ basis to develop and implement an immediate response plan. If necessary, officials will provide PEP and other treatments in the injured population. Additionally, human and animal health sector staff meet once a year to discuss rabies case reports and share prevention and control information. However, neither national nor provincial human and animal health sector officials have developed strategic and operational plans to outline the steps and actions needed to achieve the elimination of dog-mediated human rabies. The lack of a national strategic plan and a flexible “One-Health” cross-sector coordination committee were identified as major gaps.
7. Laws and regulations:
  Rabies is a notifiable human and animal disease; both human and animal health care staff must legally report identified cases within 24 hours [9]. Rabies vaccination requirements for dogs and other pets are determined according to provincial (e.g., Guizhou Province) or prefecture (e.g., Chuxiong Prefecture, Yunnan Province) level regulations. Enforcement of these laws and regulations varies by prefecture, county, and province. National level regulations for dog population management and stray or free-roaming dog rabies vaccination have not yet been developed or implemented.

**Table 2:** Common gaps, limitations, and barriers at provincial level *identified from the Stepwise Approach towards Rabies Elimination (SARE) assessment, China, March 2019.

**Data collection and analysis**
• Weak surveillance system for detecting and reporting animal rabies cases (resulting in significant under-reporting of rabies in the dog population). • Infrequent information share between human and animal health sectors.
**Prevention and control**
• Dog registration is infrequent in rural areas. • Dog rabies vaccination coverage is very low. • Lack of Integrated Bite Case Management (IBCM) at the local level
**Dog population control**
• No census data on home, stray and free-roaming dogs (nor existing methodologies or protocols). • Lack of strategic plan for dog population control.
**Laboratory diagnosis**
• Lack of laboratory diagnostic ability for animal rabies. • Difficult to obtain specimens from human rabies cases.
**Information, education and communication**
• Lack of systematic health education on rabies prevention for different sub-populations. • Inconsistent messaging on rabies prevention (including dog vaccination).
**Cross-cutting issues**
• Lack of national strategic plan towards dog-mediated rabies elimination. • Lack of “One-Health” cross-sector coordination mechanism, at national and provincial level
**Legislation**
• Lack of regulation for dog management and vaccination at national level.

* These common gaps, limitations, and barriers are listed by the seven categories outlined in the SARE assessment tool and identified by human and animal health sector representatives from the 12 provinces participating in the assessment, which included Hunan Province, Guangxi Autonomous Region, Guizhou Province, Guangdong Province, Yunnan Province, Hunan Province, Hubei Province, Hebei Province, Anhui Province, Shandong Province, Beijing Municipality and Chongqing Municipality. Provinces were selected according to the current burden of dog-mediated rabies (high, moderate, and low), interest in participating in the assessment, and availability and ability to aggregate data needed to complete the SARE tool.

### Rabies elimination in China–Next steps

Although substantial progress has been made in China on reducing the burden of human rabies, the SARE assessment identified several gaps that need to be addressed to achieve the dog-mediated human rabies elimination by 2030. Short-, medium-, and long-term goals and priority activities from the group discussions are summarized in **Table 3**. These are described by SARE category below.

1. Collection and analysis of data:
  Government officials can facilitate routine sharing of surveillance data across the human, animal, and public security sectors through official cross-agency agreements. This should include official authorization for staff from each sector to jointly collect, review, and analyze human and animal data to inform provincial and national-level decision making related to rabies elimination program progress and activities. In the long-term, developing and implementing an integrated surveillance system (including uniform standard operating procedures [SOPs]) for effectively sharing the surveillance data of human and animal rabies will be critical for achieving and documenting rabies elimination. Integration of cross-sector data using a web-based rabies surveillance database and development of a dashboard to visualize dog rabies vaccination and information on case-reports could be helpful.
2. Prevention and control of rabies:
  Development of SOPs outlining the roles and responsibilities of each sector in the prevention and control of rabies is a short-term priority. These SOPs must clearly define the roles of the human and animal CDCs as well as the role of the public security departments in dog management, particularly in rural and high-risk areas for rabies. Additional pilot-projects are needed in the medium-term to identify best practices for increasing rabies vaccination coverage in the domestic, stray, and free-roaming dog populations.
3. Laboratory diagnosis:
  In the short-term, efforts should be made by national laboratory staff to standardize the methods used for rabies diagnostic testing and communicate these methods to provincial-level laboratory personnel through training and mentoring. The national laboratory staff should also evaluate commercial rabies diagnostic kits and inform provincial laboratories accordingly. Greater collaboration and regular communication between human and animal rabies laboratories are needed in the medium- and long-term, particularly to assess rabies elimination through laboratory testing and confirmation. A key laboratory component in the elimination plan is to increase specimen collection among probable human rabies cases. Increasing provincial level capacity to collect, test, and diagnose animal rabies cases will also be critical.
4. Controlling on the number of dogs:
  A working group of experts from the human, animal, public security sectors as well as urban management and environmental sanitation and forestry should be established as soon as possible. The working group should develop, implement and monitor a strategy for improving dog population management. Also, in the short-term, a pilot dog census should be implemented in a high-risk county to assess feasibility prior to expanding to a larger area. As a medium-term activity, cross-sector training of professional staff on dog management policies and best practices should be developed and implemented. Staff at high risk of rabies exposures (i.e., contact with stray and suspected rabid dogs) should be provided with pre-exposure rabies vaccination as part of the training program.
5. Cross-department cooperation:
  The SARE assessment provided an initial opportunity for cross-sector collaboration on rabies prevention and control. International organizations–such as WHO, FAO, and OIE–described additional opportunities for cross-sector engagement through the Tripartite Guidelines [29]. China CDC recently conducted a One Health Zoonotic Disease Prioritization (OHZDP) workshop and identified rabies as a zoonotic disease of national concern [30]. Consistent with outcomes from the OHZDP workshop, a One Health working group should be established at the government level as soon as possible and include representatives from human, animal, public security, and city administration and environmental health sectors. The group should ideally meet on a quarterly basis to initially develop, and later revise, the rabies elimination strategy, as needed. Progress updates from each sector should be provided. Provincial level officials should also establish cross-sector working groups as a short-term priority activity.
6. Health promotion and rabies prevention education:
  As a short-term priority, national and provincial level staff should develop a comprehensive communication and health education plan to address each stage of the progress towards rabies elimination. The plan should develop specific approaches and materials for key populations in the high-risk provinces and target populations with varying education levels and language skills (e.g., increase use of images and videos). All communication plans should ensure consistent messaging and be disseminated through various media channels, including Weibo and WeChat. Consistent messaging should be based on scientific information for rabies prevention, use of PEP, dog vaccination and best practices for dog population management. The Chinese Center for Health Education and China CDC 12320 Health Hotline management office should engage counterparts at the provincial level as part of this short-term priority.
7. Laws and regulations:
  National and provincial representatives agreed that laws and regulations on dog rabies vaccination and management of the dog population should be made at the national level. National laws and regulations are essential for promoting and improving dog vaccination and population management related practices at the provincial and local levels. Approval of national level legislation are short- and medium-term goals.

**Table 3:** Short-, medium-, long-term goals* and priority activities identified from Stepwise Approach towards Rabies Elimination (SARE) workshop participants representing national and 12 provincial level human and animal health sector offices, March 2019. Goals and activities were identified following completion of the SARE assessment.

Short-term goals and priority activities
1. Develop and implement a national strategy on rabies elimination 2. Establish a government supported flexible ‘One Health’ multi-sector mechanism for rabies prevention and control 3. Draft provincial level work plans (including cross-sector objectives, activities, and indicators) to be reviewed and updated semi-annually 4. Integrate human rabies with animal rabies surveillance systems by establishing a shared online surveillance platform. 5. Increase the proportion of human and animal rabies cases reported to surveillance systems with laboratory confirmation by training local hospital staff on the importance and procedures for collecting specimens 6. Generate accurate estimates of the dog population 7. Develop effective approaches to control dog population by piloting local projects that can be expanded country-wide 8. Develop standard operating protocols and conduct training on IBCM (integrated bite case management) 9. Ensure sufficient supply of animal rabies vaccine by collaborating with national vaccine manufacturers 10. Develop strategies for routinely distributing audience-tailored rabies health education programs
Medium-term goals and priority activities
1. Strengthen the epidemiological investigation of human rabies cases for identifying and reporting animal rabies cases to the national animal disease surveillance system, including the development and implementation of relevant protocols and standard operating procedures 2. Ongoing implementation and evaluation of dog population management strategies 3. Improve local laboratory capacity by developing and implementing certified training programs and increasing access to necessary equipment and supplies 4. Increase the vaccination coverage to 70% by coordinating provincial and local level dog rabies vaccination campaigns 5. Update the health education strategy according to the progress of the national rabies elimination program 6. Maintain a government supported ‘One Health’ multi-sector mechanism for rabies prevention and control
Long-term goals and priority activities
1. Maintain the dog rabies vaccination coverage above 70% by ensuring availability and access to vaccine, including to dog owners in rural communities 2. Maintain efficient and high-quality surveillance for human and animal (including wild animals) rabies 3. Update the health education strategy according to progress made towards rabies elimination 4. Ongoing implementation of dog population management strategy 5. Verify the status of dog transmitted rabies elimination

*Short-term goals were identified as activities associated with Stage 1 and 2 of SARE; Medium-term goals as activities associated with Stage 3 and 4, and Long-term goals as activities associated with Stage 5.

### Improving the SARE tool

The current SARE tool consists of seven categories and 115 questions and is comprehensive and specific, and thus can play a concrete and practical role in guiding countries on the next steps towards achieving rabies elimination. However, the tool is general, and the scoring factors in each stage may not reflect the critical factors in every rabies-endemic country. Additional relevant factors could be identified and incorporated into the tool from a review of the results and feedback from countries previously completing the assessment.

In China, the affordability and appropriate use of PEP are important factors in reducing the burden of human rabies. Although mass dog vaccination is the primarily strategy for eliminating dog-mediated human rabies, appropriate and timely use of PEP remains a secondary line of defense in countries with endemic rabies. Therefore, adding two to three questions to assess the affordability and appropriate use of PEP could help improve the SARE Tool. Example questions include: 1) Is the cost of PEP fully or partial covered by the medical insurance system or by the Ministry of Health (or equivalent government agency); and 2) Does the use of PEP adhere to national and international guidelines in the rural regions of the country? In China, the New Rural Cooperative Medical System has increased the affordability of (and, therefore, access to) PEP in many rural high-risk communities. Additionally, the National Health Commission had issued revised guidelines on the use of PEP according to the WHO updated vaccine position paper [20–22], and provincial and local level CDCs have provided training of the appropriate use and timing of PEP to clinicians in rural China.

## Conclusion

This is the first time that China used a One Health cross-sector approach to assess progress towards the goal of elimination of dog-mediated rabies. The SARE tool was useful for identifying strengths and gaps in current national and provincial level rabies control and prevention programs. Human and animal health sector staff were able to collaborate in outlining program goals and priority activities. Assessment representatives agreed that a high-profile ‘Rabies Champion’ in China would help increase awareness and provide political incentives to advance rabies elimination efforts, including the development of a national strategic plan for dog-mediated rabies elimination. Rabies was prioritized as one of the top five zoonotic diseases of public concern in China during a recent One Health Zoonotic Disease Prioritization workshop. This prioritization could help promote elimination efforts using a “One Health” approach with regular cross-sectoral meetings on rabies and other priority zoonotic diseases. Assessment representatives agreed that a follow-up SARE assessment could help evaluate progress on the current short-, medium, and long-term goals and priority activities. Lessons learned should be shared with other ASEAN countries. All representatives voiced a commitment to achieving dog-mediated rabies elimination by 2030.

## Supporting information

S1 Table Summary of results for the National Rabies Program and for 12 Provincial level Programs, outlined by the seven categories outlined in the SARE assessment tool, China, March 2019. (Note: For each category, the number of pending and accomplished activities are presented. SARE referred to Stepwise Approach towards Rabies Elimination).
(DOCX)
