# Supplementary material for: Findings from the initial Stepwise Approach to Rabies Elimination (SARE) Assessment in China, 2019
Source: PLoS Negl Trop Dis. 2021 Mar 29;15(3):e0009274. doi: 10.1371/journal.pntd.0009274 (PMC8006992; doi:10.1371/journal.pntd.0009274)
Supplement: S1 Table — (Note: For each category, the number of pending and accomplished activities are presented. SARE referred to Stepwise Approach towards Rabies Elimination). (DOCX) [file pntd.0009274.s001.docx]

**S 1 Table.** Summary of results for the National Rabies Program and for 12 Provincial level Programs by the seven categories outlined in the SARE assessment tool, China, March 2019.

| Country or Province | Legislation (n=12) | | Data Collection and Analysis (n=22) | | Laboratory Diagnosis (n=13) | | Information, Education, and Communication (n=19) | | Prevention and Control (n=26) | | Dog Population Related Issues (n=12) | | Cross-cutting Issues (n=11) | |
| --- | --- | --- | --- | --- | --- | --- | --- | --- | --- | --- | --- | --- | --- | --- |
|  | Pending | Accomplished | Pending | Accomplished | Pending | Accomplished | Pending | Accomplished | Pending | Accomplished | Pending | Accomplished | Pending | Accomplished |
| China | 1 | 11 | 7 | 15 | 2 | 11 | 8 | 11 | 15 | 11 | 7 | 5 | 4 | 7 |
| Anhui | 4 | 8 | 11 | 11 | 5 | 8 | 14 | 5 | 21 | 5 | 11 | 1 | 11 | 0 |
| Beijing | 0 | 12 | 9 | 13 | 7 | 6 | 16 | 3 | 17 | 9 | 5 | 7 | 8 | 3 |
| Chongqing | 1 | 11 | 8 | 14 | 4 | 9 | 15 | 4 | 15 | 11 | 9 | 3 | 8 | 3 |
| Guangdong | 2 | 10 | 9 | 13 | 4 | 9 | 13 | 6 | 15 | 11 | 10 | 2 | 9 | 2 |
| Guangxi | 1 | 11 | 7 | 15 | 5 | 8 | 13 | 6 | 18 | 8 | 11 | 1 | 8 | 3 |
| Guizhou | 5 | 7 | 11 | 11 | 6 | 7 | 14 | 5 | 17 | 9 | 9 | 3 | 9 | 2 |
| Hebei | 2 | 10 | 9 | 13 | 9 | 4 | 13 | 6 | 19 | 7 | 9 | 3 | 6 | 5 |
| Henan | 7 | 5 | 11 | 11 | 6 | 7 | 16 | 3 | 19 | 7 | 9 | 3 | 9 | 2 |
| Hubei | 8 | 4 | 12 | 10 | 9 | 4 | 12 | 7 | 17 | 9 | 10 | 2 | 7 | 4 |
| Hunan | 3 | 9 | 7 | 15 | 7 | 6 | 9 | 10 | 15 | 11 | 10 | 2 | 5 | 6 |
| Yunnan | 2 | 10 | 10 | 12 | 5 | 8 | 12 | 7 | 16 | 10 | 9 | 3 | 8 | 3 |
| Shandong | 3 | 9 | 8 | 14 | 5 | 8 | 14 | 5 | 15 | 11 | 12 | 0 | 7 | 4 |

(Note: For each category, the number of pending and accomplished activities are presented. SARE referred to Stepwise Approach towards Rabies Elimination)
